# An outbreak of *Neospora caninum* abortion in a dairy herd from the State of Georgia, United States

**DOI:** 10.1002/vms3.346

**Published:** 2020-08-30

**Authors:** Pedro Melendez, Marcia Ilha, Moges Woldemeskel, Justin Graham, Michele Coarsey, Debi Baughman, Lisa Whittington, Hemant Naikare

**Affiliations:** ^1^ Department of Population Health College of Veterinary Medicine University of Georgia Tifton GA USA; ^2^ Tifton Veterinary Diagnostic and Investigational Laboratory College of Veterinary Medicine University of Georgia Tifton GA USA; ^3^ LeeCo Dairy Lee County GA USA

**Keywords:** abortion outbreak, dairy cattle, *Neospora caninum*

## Abstract

An outbreak of 92 abortions out of 1,700 pregnant cows (5.41%) in a period of 3 weeks (19 May to 05 June 2019) occurred in a Georgia Dairy, USA, in cattle that were between 3 and 7 months of gestation. Two sets of samples (aborted fetuses’ organs, placental tissues, aborted cows blood) were submitted for laboratory investigations at the Tifton Veterinary Diagnostic and Investigational Laboratory, College of Veterinary Medicine, University of Georgia (TVDIL, Tifton, GA, USA). An abortion panel testing for the major abortion‐causing agents [e.g. Bovine Viral Diarrhoea Virus (BVDV), Infectious Bovine Rhinotracheitis Virus/ Bovine Herpes Virus‐I (IBR/ BHV‐I), *Brucella spp., Leptospira spp*.] was conducted on several of the samples. On the first set of samples, microbial cultures, serology and PCR tests for the common abortifacient agents revealed the presence of *Neospora caninum (N. caninum)* DNA, which was positive by PCR on the placenta and fetal tissues. The second set of diagnostic investigations also identified two out of three submitted freshly aborted fetuses to be positive for *N. caninum* by PCR and immunohistochemistry. Moreover, all three dams were also sero‐positive for *N. caninum*. The entire herd was being fed on grass silage harvested from a pasture where feral pigs were hunted previously and carcasses were left behind. As a consequence of this action a large population of wild coyotes were attracted to these carcasses, and likely contaminated the pasture with potential *N. caninum*‐infected feces. After the abortion outbreak was resolved, it was recommended that the farmers should avoid disposal of cadavers of hunted animals in the wild, as it could attract carnivorous and omnivorous animals that may potentially spread the disease to the cattle and other wildlife.

## Graphical abstract

An outbreak of 92 abortions in a period of 3 weeks occurred in a 3,200 cows Georgia Dairy, USA. Two subset of samples were submitted to the Tifton Veterinary Diagnostic and Investigational Laboratory, University of Georgia. Samples were positive to *Neospora caninum* by PCR and immunohistochemistry, and dams were positive serologically to the same pathogen.

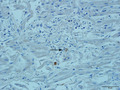

## 1 INTRODUCTION

*Neospora caninum* (*N. caninum*) is a protozoan parasite from the phylum Apicomplexa affecting several animal species, including canine, bovine, ovine, caprine, deer and rodents (Lindsay & Dubey, 2020). Neosporosis has emerged as an important reproductive disease of cattle worldwide. In cattle, it causes abortion, which was first described in dairy cows in New Mexico, USA, in 1989 (Dubey, Schares, & Ortega‐Mora, 2007). In the year 2000, a *N. caninum*‐associated abortion outbreak occurred in a dairy farm in South Carolina wherein greater than 10% of the herd aborted over a 4‐month period (Jenkins et.al, 2000). The main economic impact of neosporosis in dairy cattle is attributed to its negative impact on fertility and milk yield. The total annual cost of *N. caninum* infections/abortions was estimated to range from US $1.1 million in the New Zealand beef industry to US $546.3 million in the US dairy cattle industry (Reichel, Ayanegui‐Alcérreca, Gondim, & Ellis, 2013).

Dogs and related canids (coyotes, wolves) are definitive hosts of *N. caninum* (Klein, Barua, Liccioli, & Massolo, 2019). In addition, Australian dingoes or wild dogs (*Canis lupus dingo*) have been also identified as definitive host of *N. caninum* (King et al., 2010). The life cycle consists of three known infectious stages: tachyzoites, tissue cysts and oocysts. Tachyzoites and tissue cysts are the stages found in intermediate hosts, and they occur intracellularly (Dubey et al., 2002). The third stage (oocysts) is an environmentally resistant stage that is excreted with the faeces of definitive hosts in an unsporulated form (Dubey et al., 2007), to become invasive as early as 24 hr under room temperature of 37°C (Lindsay, Dubey, & Duncan, 1999). The mechanism of survival of *N. caninum* oocysts in the environment is not well understood. One of the main routes of infection for carnivores and omnivores is the ingestion of tissue cysts being presented in muscles of their prey (Lindsay & Dubey, 2020). Herbivores are likely to become infected by the ingestion of food or drinking water contaminated by *N. caninum* sporulated oocysts (Dubey et al., 2007). Ingestion of faecal‐contaminated food and water is the major source of infection for cattle that may cause an epidemic abortion storm in naïve population, after transplacental transmission of *N. caninum* to the fetus (Qian et al., 2017). This paper reports an abortion outbreak associated with *N. caninum* infection in a large Holstein herd from South Georgia, USA.

## 2 CASE DESCRIPTION

### 2.1 Farm

The outbreak occurred in a dairy farm located in Leesburg, GA, USA (Latitude: 31.68; Longitude: −84.17), which consisted of 3,400 lactating Holstein cows, housed in a free‐stall system with sand‐bedding, fans and sprinklers. Cows were milked three times a day with an average milk yield of 38.6 kg cow^−1^ day^−1^, 190,000 somatic cell counts and standard plate bacteria count of 1,300 cfu.

Lactating cows were fed three times a day a total mixed ration based on grass silage, corn silage and concentrates. Forages were produced in the same farm.

Right after drying‐off, cows were sent to another farm to give birth. Fifteen days after parturition, cows were moved back to the original farm. Reproductive management started at 40 days post‐partum (synchronization of oestrus protocols) and first breeding occurred around 70 days in milk. Pregnancy diagnosis was performed by the local veterinarian by ultrasound at 32 days’ post artificial insemination. Cows were reconfirmed to be pregnant by rectal palpation between 50 and 70 days’ post service. If cows were found non‐pregnant, they were resynchronized and bred back. After 70 days of pregnancy cows were moved to a pregnant group until dry‐off. Historically pregnancy losses (abortion rate) that occurred from 70 days of gestation until drying‐off (7 months of gestation) at this dairy farm were less than 1% per month.

The herd was strategically vaccinated before breeding with a modified live virus commercial vaccine against infectious bovine rhinotracheitis virus (IBRV), bovine viral diarrhoea virus (BVDV; type 1 and 2), parainfluenza 3 (PI_3_) virus and bovine respiratory syncytial virus (BRSV) plus a 5‐way vaccine against *Leptospira icterohaemorrhagiae* (VISTA^®^ 5 L5 SQ; Merck & Co., Inc., Kenilworth, New Jersey, USA) and a 7‐way commercial vaccine against clostridial diseases (Vision^®^ 7 with SPUR^®^; Merck & Co., Inc., Kenilworth, New Jersey, USA).

### 2.2 Abortion outbreak description

Overall, 92 abortions occurred in a span of 18 days (19 May 2019 and 5 June 2019) in cattle that were between 3 and 7 months of gestation. These abortions occurred within a population at risk consisting of 1,700 pregnant lactating cows, accounting for an incidence of 5.45%.

Table 1 illustrates the timeline of the abortion storm. A detailed description of the outbreak is as follows: During the week of 19th–25th May 2019, 18 cases of abortion from different barns of the dairy were recorded. Gestational age of fetuses was between 100 and 212 days. Interestingly, 17 out of 18 aborted cows were from parity 1. Necropsies of aborted fetuses were conducted in the field by the farm veterinarian. Blood samples from five aborted cows, remains of fetal membranes from three aborted cows, and organ biopsies from two aborted fetuses were submitted for disease investigation to the Tifton Veterinary Diagnostic and Investigational Laboratory (TVDIL) of the University of Georgia. Between 26th and 28th May 2019 another 42 abortion cases were identified, with a gestational age between 81 and 205 days from cows within parity 1 and 5. Three fresh fetuses and blood samples from these aborted cows were submitted to the TVDIL. In the week of 29th May to 4th June 2019, another 31 abortion cases were diagnosed. Gestational age of fetuses and parity number of aborted cows ranged from 89 to 237 days, and 1 to 4, respectively. Drastically, the number of abortion decreased, with only one abortion identified on 5th June (250 days of gestational age from a cow of parity 2). No samples were received at the TVDIL for laboratory investigations from abortions that occurred between 29 May and 5 June 2019. Also, no samples were received during the period from 6 to 12 June 2019. After receiving of the preliminary laboratory results, the dairy field‐service veterinarian (first author of this case presentation report) from the TVDIL, visited the dairy farm on 13 June 2019. From 5th June until the day of farm visit, no more noticeable abortion cases were identified.

**TABLE 1:** Timeline of the abortion outbreak

Period	No. abortions	Gestational age of fetus (days)	Parity of cow	Samples received at the TVDIL
19 May 2019 – 25 May 2019. (7 days)	18	100–212	1 (17 out of 18 cows)	Organ biopsies from 2 aborted fetuses, placental tissues from 3 aborted cows and blood samples from 5 aborted dams
26 May 2019 – 28 May 2019. (3 days)	42	81– 205	1 to 5	3 fresh fetuses, 1 placental tissue and blood samples from their dams
29 May 2019– 4 June 2019. (7 days)	31	89–237	1 to 4	none
5 June 2019. (1 day)	1	250	2	none
6 June 2019 – 13 June 2019. (8 days)	0	not applicable	not applicable	not applicable

Records from the dairy computer systems were obtained. A walk through of all lactating and dry‐cow barns was carried out and the cows were visually inspected. Cows were observed to have a moderate to excellent body condition score, ranging from 2.75 to 3.25 on a scale 1 to 5 (Ferguson, Galligan, & Thomsen, 1994). They appeared to be very clean and were using almost 95% of the sand bedded free‐stalls. Faeces showed very good consistency and almost 65% of laying cows was ruminating.

Samples from the corn silage, grass silage and total mixed ration were submitted to the TVDIL to check the presence of *N. caninum* DNA.

### 2.3 First set of diagnostic investigations

#### 2.3.1 Materials and Methods

Blood samples from five aborted cows, placental tissues (cotyledons and membranes) from three aborted cows, and sections of liver, spleen, kidneys and lungs from two aborted fetuses (abortions from the week of 19–25 May 2019) were received at the TVDIL for the first round of investigation. Representative tissue samples were fixed in neutral‐buffered 10% formalin and processed for routine microscopic examination, stained with haematoxylin and eosin (H&E) and examined under light microcopy. In addition, abortion panel testing that comprised of bacterial culture, virus isolation and fluorescent antibody tests were performed on fetal membranes from aborted cows and organs from aborted fetuses. The fresh tissues were also evaluated by PCR tests (probe‐based Taqman real‐time qPCR assays; Barry et al., 2019; Brower et al., 2008) for the presence of DNA of abortifacient infectious agents: *Leptospira* pathogenic serovars, *Tritrichomonas foetus*, *Campylobacter fetus*, *Anaplasma marginale*, BVDV, Bovine Herpes virus‐1&4, *Salmonella* spp., *Chlamydophila* spp*, Listeria monocytogenes, Coxiella burnetii* and *N. caninum*. All microbiological testing and histopathology based testing (Table 2) were performed in accordance with the approved standard operating procedures of the quality system at the TVDIL with appropriate controls for each assay. The table 2 provides a summary of diagnostic methods utilized and the corresponding test results that were derived from both sets of laboratory investigations.

**TABLE 2:** Summary of diagnostic methods & test results from laboratory investigations

Diagnostic Method employed for microbial detection	Assay (TVDIL In‐house protocol/ commercial) & Reference	Clinical specimens tested	Results
Routine & special cultures for bacterial and fungal isolation & identification	In‐house protocol for aerobic and anaerobic bacteria; special cultures for *Brucella Campylobacter*, Fungal, *Listeria;* Quinn (1994)	Set #1: Fetal tissues from 2 fetuses, placenta from 3 aborted cows, blood samples from 5 dams; Set#2: Fetal tissues from 3 fetuses, one placenta, 3 blood samples from dams	No significant bacteria identified from neither sets of tissues upon routine culture and special cultures
Microscopic Agglutination Test (*Leptospira*)	In‐house protocol; WOAH OIE Terrestrial Manual (2019)	Serum from dams from set #1 and set #2	Negative
Serum Neutralization (SN) Assays (BVD−1a, 1b & 2; IBR (BHV−1) & BHV−4	In‐house protocol; Lennette etal (1979)	Serum from dams from set #1 and set #2	SN tests were positive for BVD−1a, 1b & 2; IBR (BHV−1) from all dams in set #2; SN test for BHV−4 was positive from one dam serum from set #2
Fluorescent Antibody Tests (BVD, IBR, BTV, *N. caninum*)	In‐house protocol Kawamura (1977)	Serum from dams from set #1 and set #2	Negative
ELISA testing (BVD; *Anaplasma, Leptospira*, BTV, *N. caninum*)	Commercial kits *Anaplasma* Ab, BTV Ab, *N. caninum* Ab kits (VMRD Inc.); BVD Kit (Idexx)	Serum from dams from set #1 and set #2; body cavity fluids from fetuses from set #2	*N. caninum* ELISA was positive on sera from all dams from set #2; BTV Elisa was positive in sera from 2 dams from set #2 BVD ELISA result was ‘suspect’ on fetus no. 2
Virus isolation	In‐house protocol (Madin‐Darby Bovine Kidney cell line)	Blood samples (buffy coat) from set #1 and # 2	Negative
Nitrate Toxicity	In‐house protocol	Ocular fluid from two fetuses from set#2	Negative
Immunohistochemistry (IHC) for *N. caninum*	PAB‐NC antiserum (VMRD Inc.)	Fetal tissues from set #2	Positive on tissues from fetus no. 1 and 2.
Necropsy and Histopathology	In‐house protocol	Tissues received under set #1 and set #2; Fetuses (no.1, 2 and 3) from set #2	No significant lesions observed on haematoxylin‐eosin stained slides of fixed tissues from set #1; Mild multifocal non‐suppurative encephalitis with glial nodules in brain of fetus no.1 of Set #2. No other significant lesions in other fetal tissues from Set #2.
**Molecular Testing** (2‐step: Nucleic acid extraction and PCR amplification for abortifacient pathogen detection)	Nucleic acid extraction with QIAamp DNA mini kit and Rneasy mini kit (Qiagen); PCR amplification: In‐house & commercial kits	Placenta & fetal tissue DNA & RNA from set #1 and set #2	See below
*Leptospira spp*.	LSI‐Vet Max TFS kit	Listed above	Negative
*Anaplasma marginale*	In‐house protocol	Listed above	Negative
*Tritrichomonas foetus*	In‐house protocol	Listed above	Negative
*Campylobacter foetus*	LSI‐Vet Max TFS kit	Listed above	Negative
*BVDV*	Commercial kit (TFS)	Listed above	Negative
*BHV−1 &BHV−4*	In‐house protocol	Listed above	Negative
*Chlamydophila*	LSI‐Vet Max TFS kit	Listed above	Negative
*Listeria monocytogenes*	LSI‐Vet Max TFS kit	Listed above	Negative
*Coxiella burnetii*	LSI‐Vet Max TFS kit	Listed above	Negative
*Neospora caninum*	Two In‐house protocols: 1) Barry *etal. Vet. Parasitol* (2019); 2) Brower *etal. JVDI* (2008)	Listed above	Positive by both protocols for all nucleic acid extracts yielding Ct values between 30 and 34 in a 40 cycle PCRs

#### 2.3.2 Results

No significant lesions were observed on histopathological examination in the sections of placenta from the three aborted cows as well as liver, lungs, spleen and kidneys from the two aborted fetuses. Fluorescent antibody tests for BVDV, IBR virus, Blue Tongue virus (BTV) and *N. caninum* were negative. Similarly, virus isolation in all tissues was negative. Mixed bacterial organisms (contaminants) were present in the examined tissues on culture. PCR testing for all abortifacient infectious agents (listed above) were negative except *N. caninum*. All examined tissues including placenta from the cows and tissues from aborted fetuses were positive for *N. caninum* by PCR.

### 2.4 Second set of diagnostic investigations

#### 2.4.1 Materials and Methods

Three fetuses and one placental tissue (abortions from 26 to 28 May 2019) were submitted to the TVDIL for necropsy (fetuses 1, 2 and 3) along with samples of blood from the dams. During necropsy, samples were collected for histopathology, bacterial cultures, serology and virology and PCR testing (probe‐based Taqman real‐time qPCR assays; Barry et al., 2019; Brower et al., 2008) (Table 2). In addition, ocular fluid from fetuses 1 and 2 were collected for nitrate test. Serology for the following infectious agents was performed on the serum from the dams: BTV, *Leptospira spp*., *Anaplasma spp*., *N. caninum*, BVDV types 1a, 1b and 2, IBRV and BHV‐4. In addition, virus isolation was attempted on the buffy coat of dam's blood.

#### 2.4.2 Results

**FIGURE 1:**
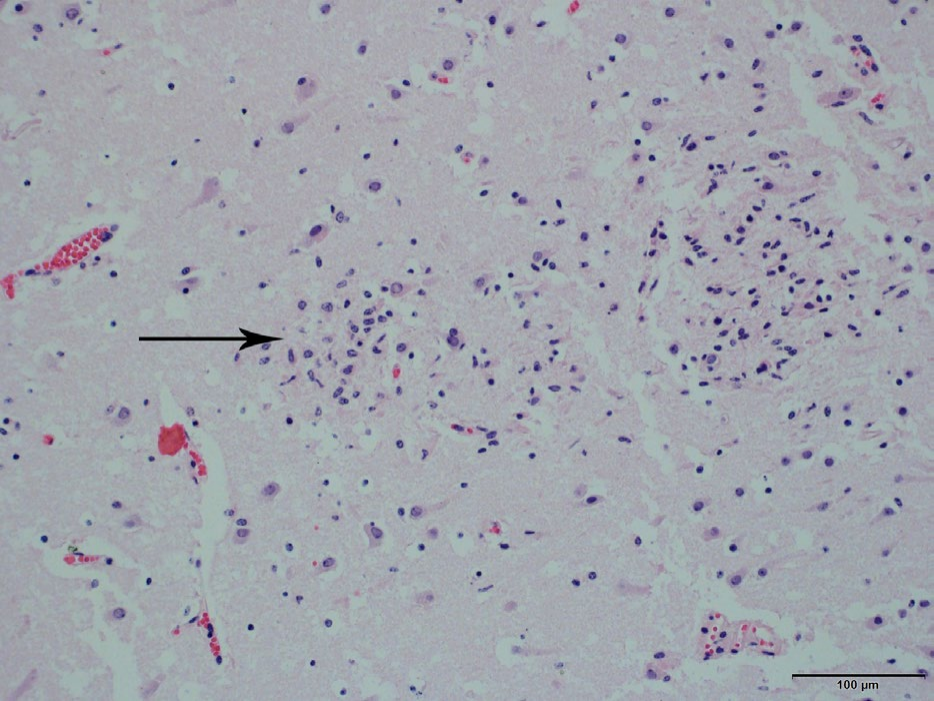
Fetus 1. Brain. Multifocal glial nodules (arrow). HE stain

Fetus 1 was a female, 12 kg, 60 cm long (crown‐rump length, approximately 205 days of gestation) Holstein bovine fetus. On necropsy, there were oedema of subcutaneous tissue and approximately 200 ml of serosanguineous fluid in the thoracic cavity. On histopathological examination, mild multifocal lymphohistiocytic myositis and myocarditis were observed in tongue, skeletal muscle and heart. Mild multifocal non‐suppurative encephalitis with glial nodules was observed throughout the brain (Figure 1).

Fetus 2 was a male, 3 kg, 36 cm long (crown‐rump length, approximately 145 days of gestation) bovine fetus. The fetus was submitted frozen. There were approximately 5 ml of serosanguineous fluid in the abdomen and 5 ml of serosanguineous fluid in the thoracic cavity. Significant lesions were not observed on histopathology.

Fetus 3 was a male, 256 grams, 15 cm long (crown‐rump length, approximately 81 days of gestation) bovine fetus. Placenta was also received. The fetus and placenta were presented frozen. There were no significant necropsy findings. On histopathology, fetal tissues and sections of placenta had marked freezing artefact and were autolysed. Despite autolysis, random infiltrates of neutrophils and macrophages were observed in placental sections.

The histopathological lesions in the brain of fetus 1 were highly suggestive of *N. caninum* infection. PCR for *N. caninum* was performed in pooled tissue samples from each individual fetus in all three fetuses. PCR results were positive for *N. caninum* in all three fetuses. No other PCR tests for abortifacient infectious agents were positive. BVDV ELISA was negative in fetus 1, resulted suspect in fetus 2 and was not performed in fetus 3. Aerobic and anaerobic bacterial cultures did not yield any significant growth in tissue samples from all three fetuses. In addition, *Brucella, Campylobacter*, *Salmonella, Tritrichomonas foetus* and *Listeria* cultures were negative in all three fetuses. Nitrate test on ocular fluid was negative in fetuses 1 and 2. Cavity fluids from fetuses 1 and 2 collected during necropsy resulted negative for the following serological tests: BTV antibody ELISA, *Leptospira* antibody MAT, *Anaplasma* ELISA and *Neospora* ELISA.

Interestingly, *N. caninum* ELISA was positive from sera of all three cows. In addition, BTV antibody ELISA was positive in two cows and BHV‐4 antibody serum neutralization was positive in one cow. All cows were positive for IBR and BVDV types 1a, 1b and 2 by serum neutralization. Seropositive results indicate prior exposure and/or response to immunization. Virus isolation on samples of blood buffy coat was negative in all three cows.

**FIGURE 2:**
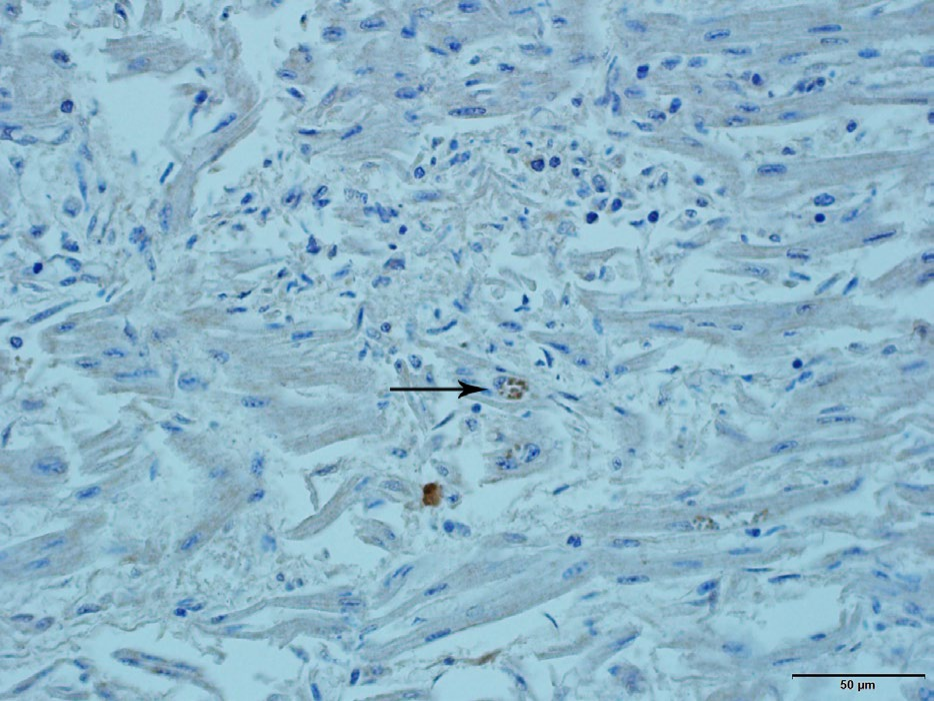
Fetus 2. Heart. Multifocal positive immunostaining for *Neospora caninum* (arrow)

To further confirm diagnosis, immunohistochemistry (IHC) for *N. caninum* (PAB‐NC, VMRD Inc., Pullman WA) was performed in tissue sections of brain, skeletal muscle and heart from fetus 1 and tissue sections of brain, tongue and heart from fetus 2. Positive immunostaining for *N. caninum* was observed in sections of skeletal muscle and heart from fetus 1 and sections of heart from fetus 2 (Figure 2).

## 3 DISCUSSION

This herd's normal abortion rate in cows over 70 days of gestation was less than 1% per month prior to 19 May 2019. The abortion rate escalated to 5.41% in a period of 3 weeks (19 May–5 June 2019). Unquestionably, this spike in fetal losses within a short period of three weeks classifies this case as an ‘abortion storm’ or ‘outbreak’. A disease outbreak is the occurrence of disease cases in excess of normal expectancy. The number of cases varies according to the disease‐causing agent, and the size and type of previous and existing exposure to the agent (WHO, 2019). The increase in abortion cases led the local veterinarian to submit aborted fetuses, placenta tissues and blood samples from affected cows to the TVDIL.

Histopathological findings in aborted fetuses, immunohistochemistry, *N. caninum* positive PCR results and *Neospora* positive serological results from aborted cows strongly suggested that the causative agent of the current outbreak was *N. caninum*. Detection of *N. caninum* specific DNA by PCR is a highly sensitive method for the identification of *N. caninum* in aborted fetal tissues, particularly for a probe‐based Taqman real‐time qPCR assay that offers an accurate and rapid detection of the parasite from contaminated tissues. The qPCR results were confirmed by two different assays (Barry et al., 2019; Brower et al., 2008) and the positive results were in 100% agreement. Both assays consistently yielded Ct values between 30 and 34 in 40 cycle PCR runs for all tissue DNA extracts further confirming the presence of *N. caninum* DNA. However, the contribution of other infectious agents (e.g. BVD virus as immunosupressor) and undetected risk factors cannot be ruled out. While one of the laboratory results showed evidence of positive serology to BVD virus type 1b from aborted cows, at the same time the herd was carrying out routine vaccination against viral diseases. Similarly, the significance of positive BTV serology test results cannot be determined, as BTV antibodies are commonly seen in the bovine sera that are received at the TVDIL. Notably, exposure to the BTV under natural conditions in dairy herds has been associated with an increase in the occurrence of abortions, regardless of the stage of pregnancy (Nusinovici, Seegersa, Joly, Beaudeaua, & Fourichon, 2012). In addition, positive serology to one of the aborted cows to BHV‐4, also raises a question about the role of this herpesvirus in the present outbreak investigation, because this virus has been isolated from several epidemic events of metritis and abortions. BHV‐4 has also been considered as a pathogen associated with uterine disease in dairy cattle (Donofrio et al., 2007).

When the positive results of *N. caninum* infection were confirmed, the local veterinarian proceeded with an inspection of the pastures where grass was harvested for silage. Looking for source of infection, the local veterinarian searched for canid feces on the farm. Further discussions with the farm manager revealed that as a control measure to an invasion of wild pigs in the pastures where these cows were managed, a company that specialized in the control of pests to eliminate wild hogs was hired. It was determined that several carcasses of the slaughtered pigs remained exposed on the pastures where the presence of a large number of coyotes was subsequently observed consuming the remnants of hog carcasses. The PCR examination of feed did not confirm the presence of *N. caninum* DNA.

The most common way of transmission of *N. caninum* in cattle is by the ingestion of faecal contaminated feed and water that may cause an epidemic abortion storm in naïve cattle population, after transmission to the fetus (Qian et al., 2017). Consequently, the concentration of coyotes potentially infected with *N. caninum*, in a reduced area where grass was used as feed (silage) for lactating cattle, strongly suggests that the faeces of these canids could easily infect the pastures of this field and transmit the parasite to cattle. Interestingly, in a recent study conducted in China, it was found that the overall seroprevalence of *N. caninum* in several swine farms was 1.9% (range from 0.3% to 4.6%). In addition, DNA was extracted from 600 brain samples, and three (0.5%) of the examined brains were found to be positive to *N. caninum* DNA, providing molecular evidence that pigs are also a natural intermediate host of *N. caninum,* and may play an important role in the epidemiology of the disease (Gui et al., 2020). Furthermore, in the USA, white‐tailed deer are similarly an important intermediate host for maintaining the cycle of *N. caninum*, where urbanized environments and deer density may facilitate the transmission of the parasite from domestic animals (dogs) to wildlife (deer, feral pigs, coyotes). Indeed, in a study conducted in the state of Ohio, 23.6% (105/444) of white‐tail deer were seropositive to *N. caninum,* where adult deer from urban areas were at greater odds of being seropositive than those from country side habitats (Ballash et al., 2019). Moreover, in Calgary, Alberta, Canada, was found that 10% of sampled coyotes from five city parks were positive to detectable amounts of *N. caninum* DNA. The authors of this investigation also stated that white‐tailed deer and mule deer were common in Calgary's city parks, indicating that the presence of *N. caninum* DNA in coyote faecal samples was indicative that a sylvatic host of *N. caninum* exists within this urban environment (Klein et al., 2019). Taking into consideration the aforementioned, it is reasonable to suggest that the permanence of the carcasses of hunted pigs on the pastures, and the presence of deer observed commonly in this dairy could concentrate the coyote population in a reduced area. This could generate an ideal environment for the complete cycle of *N. caninum* and faecal contamination of the pastures used to produce silage, that subsequently infected the naive population of cows from this dairy, triggering the abortion storm.

On the basis of the recent studies cited above, the manager of the farm inspected in detail the field where coyotes were observed in a large number. Unfortunately, faecal material from the canids was not found, recalling the grass from this field was previously harvested for silage production. After the abortion outbreak was resolved it was recommended that the owners of the farm not leave cadavers of hunted animals, which may serve as food for coyotes or other carnivores and omnivores that may potentially spread the disease.

In conclusion, an abortion storm in a large confined dairy herd from Georgia, USA occurred in a period of 3 weeks. A thorough laboratory diagnostic investigation of several samples (aborted fetuses, placentas, blood from aborted dam) led to the following findings: *N. caninum* intermediary‐stages of the parasite, DNA of the parasite and dam's serum antibodies. These results suggest that the most likely aetiological source for abortion cases in this naïve cattle population was *N. caninum*.

## 4 ETHICS STATEMENT

The authors confirm they followed the ethics policies of the journal and the University of Georgia.

## CONFLICT OF INTEREST

The authors have no conflict of interest to declare.

## AUTHOR CONTRIBUTION

**Pedro Melendez:** Formal analysis; Investigation; Writing‐original draft. **Marcia Ilha:** Conceptualization; Investigation; Methodology; Validation. **Moges Woldemeskel:** Conceptualization; Investigation; Methodology; Validation. **Justin Graham:** Conceptualization; Investigation; Resources. **Michel D Coarsey:** Formal analysis; Methodology. **Debi McLendon:** Formal analysis; Methodology. **Lisa Whittington:** Formal analysis; Methodology. **Hemant Naikare:** Conceptualization; Formal analysis; Investigation; Methodology; Resources; Validation; Writing‐review & editing.

### PEER REVIEW

The peer review history for this article is available at https://publons.com/publon/10.1002/vms3.346.
